# A Singular Case in Practice

**Published:** 1877-04

**Authors:** A. O. Rawls


### A SINGULAR CASE IN PRACTICE.

BY A. O. RAWLS, D. D. S.

Mrs. R———, called January 24th, for the purpose of having an inferior right second bicuspid removed, filled and replanted. Previous to her calling, the tooth had been giving her much pain, so much indeed that her naturally nervous condition had been wrought up to an unusual degree of nervous excitement. Upon examination I found the tooth presenting all the usual signs of chronic periostitis. There was quite a large decayed cavity extending from posterior approximal surface into the pulp cavity. A small portion of nerve tissue was in the root canal, which seemed to be but partially vitalized. Chloroform was administered to the patient, though on account of her weakened condition, not to complete anæsthesia. The tooth extracted and placed in a vessel of warm salt water, I then turned my attention to the patient until she recovered from the effects of the chloroform, which was but a few minutes, after which I saturated a small napkin with the warm salt water, took the tooth out of the water and wrapping the root about in the napkin, stepped to my White's engine and began burring out the decay; the very instant that the bur touched in the cavity the patient screamed with pain; I ceased working with the tooth immediately and asked the patient what caused her to scream so. She informed me that I had been cutting into that tooth, and that it pained her the same as if it had been in her mouth. I thought it but a coincidence, and began with the bur again, which caused her to scream again, and throughout the operation of cleansing the decayed cavity, she evinced signs of pain, especially when the instrument touched a point near the junction of the enamel and dentine. Having finished excavating the decay I walked to a position in the rear of my operating chair where it was impossible for the patient to see me without getting up and turning around. I then took an ordinary barbed nerve broach, inserted it gently into the nerve cavity and in the same instant that the instrument touched the dead nerve, my patient jumped up in the chair with a shriek as if in the most violent pain, exclaiming, "You are touching the nerve." I then determined to test more thoroughly the matter of coincident pain in the jaw with the touch of an instrument to the nerve, and accordingly turning my face from the patient, and holding the tooth in a position which would make it impossible for her to see or know what I was doing with it. I touched the nerve two or three times in quite rapid succession, and found that she evinced the same signs of pain each time. I waited a few minutes and pushed the nerve broach about half the length of the nerve canal, when she screamed again, plunging the broach a little deeper and giving it a twist I was enabled to remove it, but not without another manifestation of pain from the patient. After removing the nerve I proceeded to syringe the pulp cavity and nerve canal with cold water, the patient knew nothing of what I was about to do, neither did she see or know that I had a syringe in my hand, but immediately on the introduction of cold water in the tooth she not only shrieked with pain, but made all the usual signs that follow the sudden forcing of a stream of cold water into the mouth and upon sensitive dentine, and informing me immediately that I was putting water in the tooth, and cold water too. I then requested her to step into the reception room and going with her engaged herself and husband in conversation on topics entirely foreign to dentistry. After the lapse of some ten or fifteen minutes I returned to my operating room, leaving the patient and her husband conversing together in the waiting room. Securing a sharp hoe excavator, and taking a position where there would intervene between myself and patient a brick partition wall, I selected a point in the decayed cavity of the tooth, immediately beneath one of the cusps, and after poising the instrument for a moment over this point I gave a quick sharp cut into the dentine and was greeted with another shrill cry from my patient in the next room, and again told by her that "I was cutting that tooth." Previous to cutting the dentine however I listened and noted carefully that she and her husband were in conversation and am certain that she did not hear the instrument cut or know what I was about to do. A few minutes after I proceeded to fill the tooth according to my usual method and placed it back into its former position in the mouth. Have seen the patient twice since and found the tooth looking as well as could be expected.

